# Infection, Screening, and Psychological Stress of Health-Care Workers With COVID-19 in a Nonfrontline Clinical Department

**DOI:** 10.1017/dmp.2020.428

**Published:** 2020-11-04

**Authors:** Ge Wang, Jia-Lun Guan, Xiu-Qing Zhu, Mu-Ru Wang, Dan Fang, Yue Wen, Meng Xie, De-An Tian, Pei-Yuan Li

**Affiliations:** Division of Gastroenterology, Tongji Hospital, Tongji Medical College, Huazhong University of Science and Technology, Wuhan, China

**Keywords:** COVID-19, health-care worker, nonfrontline, psychological stress, risk factors

## Abstract

**Objectives:**

The aim of this study was to investigate risk factors and psychological stress of health-care workers (HCWs) with coronavirus disease 2019 (COVID-19) in a nonfrontline clinical department.

**Methods:**

Data of 2 source patients and all HCWs with infection risk were obtained in a department in Wuhan from January to February 2020. A questionnaire was designed to evaluate psychological stress of COVID-19 on HCWs.

**Results:**

The overall infection rate was 4.8% in HCWs. Ten of 25 HCWs who contacted with 2 source patients were diagnosed with confirmed COVID-19 (8/10) and suspected COVID-19 (2/10). Other 2 HCWs were transmitted by other patients or colleagues. Close care behaviors included physical examination (6/12), life nursing (4/12), ward rounds (4/12), endoscopic examination (2/12). Contacts fluctuated from 1 to 24 times and each contact was short (8.1 min ± 5.6 min). HCWs wore surgical masks (11/12), gloves (7/12), and isolation clothing (3/12) when providing medical care. Most HCWs experienced a mild course with 2 asymptomatic infections, taking 9.8 d and 20.9 d to obtain viral shedding and clinical cure, respectively. Psychological stress included worry (58.3%), anxiety (83.3%), depression (58.3%), and insomnia (58.3%).

**Conclusions:**

Close contact with COVID-19 patients and insufficient protection were key risk factors. Precaution measures and psychological support on COVID-19 is urgently required for HCWs.

On December 2019, cases of viral pneumonia of unknown etiology occurred in Wuhan, Hubei Province, China. The etiology was identified to be a novel coronavirus and subsequently renamed as severe acute respiratory syndrome coronavirus 2 (SARS-CoV-2) on February 11, 2020.^1,2^ Subsequently, rapid increased cases with similar clinical characteristics and contacting history confirmed that SARS-CoV-2 can transmit between people.^2,3^ The World Health Organization (WHO) stressed its risk by declaring this disease as a Public Health Emergency of International Concern (PHEIC) on January 30, 2020.^4^ As of June 10, 2020, there were 84,641 confirmed COVID-19 cases in China, and 7,060,898 coronavirus disease 2019 (COVID-19) cases across other 200 countries and territories.^5^

SARS-CoV-2 can spread through droplets and contact, not excluding aerosol transmission and fecal-oral transmission. People are generally susceptible.^6^ Because of poor knowledge about COVID-19 and its strong infectivity, some health-care workers (HCWs) were reported to be infected with SARS-CoV-2 in the early stage.^7^ Data showed more than 3000 HCWs have been diagnosed with COVID-19 in China.^8^ The infection situation of HCWs abroad is also very serious. Between March 16 and March 29, 2020, a total of 1533 symptomatic HCWs who worked in a hospital in United Kingdom were tested, of whom 282 (18%) were positive for SARS-CoV-2.^9^ As of March 27, more than 5000 HCWs in Italy have been infected and 41 HCWs have died from COVID-19.^10^ In America, the infected HCWs have been more than 9000 in early April and of whom 27 have died.^11^ Announced by WHO on April 8, the COVID-19 pandemic has hit over 22,000 HCWs across 52 countries and regions, and this number was probably underestimated.^12^ The fact that HCWs are at risks of infection in the epidemic chain is a critical issue because HCWs help in preventing and controlling the outbreak. Uncontrollable transmission in medical facilities not only worsens personnel shortage, but more importantly potentiates virus spread.

HCWs in the frontline, such as fever clinic, emergency department, respiratory medicine department, infections department, and designated hospital, are supposed to be at high risks of SARS-CoV-2 infection. Nevertheless, the investigation of SARS-CoV-2 transmission among HCWs outside the frontline department was rare but important. However, data on demography, risk factors, and infection process of HCWs with COVID-19 are lacking, especially in health-care settings.^6,7^ Thus, we here report a case series of SARS-CoV-2 infections in HCWs at early stage of the outbreak from a nonfrontline department in a hospital in Wuhan, China.

## Methods

### Patients

Two source patients onset with abdominal symptoms were admitted to the department of gastroenterology in a hospital in Wuhan, China, from January 6 to January 29, 2020. Screening for COVID-19 was quickly performed in these patients when they had a fever. Subsequently, 12 ill HCWs in the same department were monitored, and most of them were identified as having COVID-19 according to current guidance.^6^ Oral consents were obtained from all patients enrolled. The investigation was approved by the Institutional Ethics Board of Tongji Hospital. All infected HCWs were followed up from January 20 to May 20, 2020.

### Data Collection

Data on epidemiology, screening, quarantine, treatment of source patients and 12 infected HCWs were recorded. Electronic medical records, phone calls, and an online questionnaire survey were used to obtain the data. The medical care behaviors and occupational protection were retrospectively studied from 12 HCWs. A suspected COVID-19 case includes any person presenting with symptoms of acute respiratory tract infection or other clinical illness compatible with COVID-19, or an asymptomatic person who has close contact with a confirmed case. A confirmed COVID-19 case is one with positive results of the nucleic acid test, or genomic sequence, or specific serological antibodies against SARS-CoV-2.^6^ The durations from illness onset to hospital admission or designated isolation, and from illness to clinical recovery or viral shedding of SARS-CoV-2, were calculated, respectively.

Furthermore, an online questionnaire survey was designed to evaluate the psychological stress of the outbreak on HCWs. The subjective feelings of anxiety, depression, and the symptom of insomnia were collected as none, mild, medium, or severe. The considerations on COVID-19 were questioned as “How do you think about COVID-19 before infection?” and “How do you think about COVID-19 after infection?”. The options designed for questions on impression of COVID-19 including following items: just like a heavy influenza, I will be fine because I am young, worrying about long lasting, worrying about aggravation, worrying about life threatening, worrying about difficult viral shedding of SARS-CoV-2, worrying about relapse, and worrying about transmission to families. The confidence to continue working in the health-care profession, and the consideration on occupational protection manner in the future clinical practice were also asked.

### Data Analysis

Data were presented with mean ± SD, median (interquartile range), or number (percentage).

## Results

### Epidemiology, Infection, and Contact Tracing of Source Patients and HCWs with COVID-19

Source patient 1, a 32-year-old man, presented with abdominal pain and hematochezia on January 14, 2020, as well as fever of 38.5°C once. The CT images showed sporadic ground-glass opacity (GGO) located in bilateral lung lobes. The patient was then rapidly quarantined in an isolation ward because of suspected COVID-19 infection on the next day of admission.

Source patient 2, a 45-year-old woman, was hospitalized for recurrent abdominal pain and vomiting on January 6, 2020. She had a fever on January 29. With obvious imaging features of pneumonia and positive nucleic acid test of SARS-CoV-2, the patient was confirmed with COVID-19 and transferred to the isolation ward.

**Figure 1.**
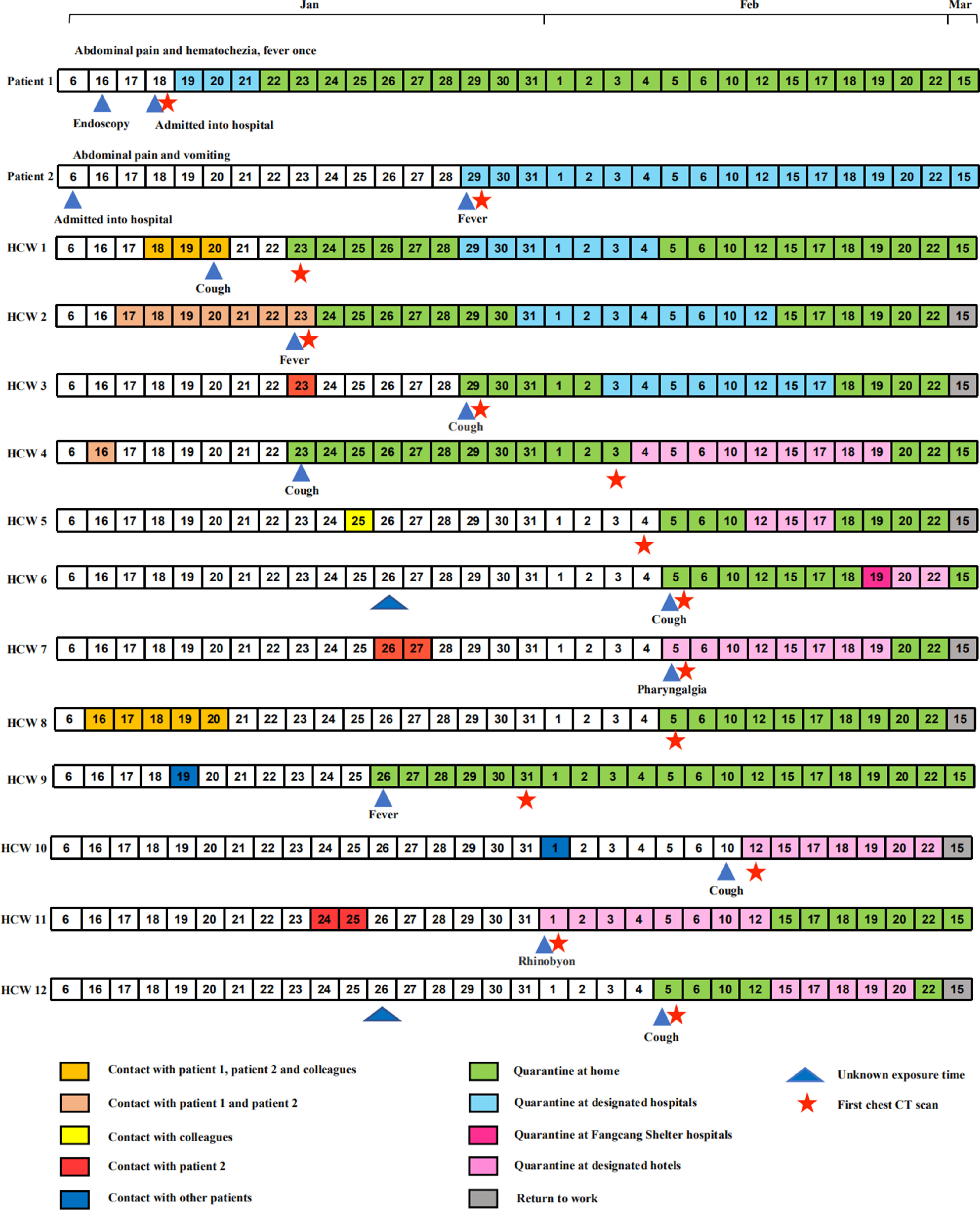
Chronology of symptom onset of 12 infected HCWs and their contacts with suspected COVID-19 patients. Source patient 1, clinically identified with COVID-19, and source patient 2, laboratory-confirmed COVID-19, were admitted to the hospital on January 18, 2020, and January 6, 2020 (white box), respectively. Close contacts were marked with boxes of different colors. Days of exposure were presented with consecutive numbers of boxes with different colors. HCW 6 had close contacts with patient 2 and colleagues. HCW 12 had close contacts with patient 1, patient 2, and colleagues, with unknown exposure time. Their first chest CT scan were performed quickly. HCWs 1-10 were confirmed COVID-19 cases, and HCWs 11-12 were suspected COVID-19 cases. Most of HCWs were isolated at home from symptoms onset (arrow). Some were quarantined in designated hotels or Fangcang Shelter hospitals. Three HCWs and 2 source patients were hospitalized. HCW 5 and HCW 8 were of asymptomatic infection. In March, some HCWs recovered and returned to work. HCW, health-care worker; CT, computed tomography.

During their hospitalization in the department of gastroenterology, 25 HCWs have ever contacted them closely. Ten of them (40%) were then diagnosed with viral pneumonia, with 8 confirmed COVID-19 cases and 2 suspected COVID-19 cases, respectively. There were 2 more HCWs with confirmed COVID-19, who were not directly related to these 2 source patients. A comprehensive chronology of symptom onset of the 12 HCWs and their contacts with suspected COVID-19 cases was described (Figure 1; Table 1). Except HCW 4, HCW 9, and HCW 10 worked in the endoscopic center, others worked in the ward (HCW 10 worked in the fever clinic temporarily under secondary protection equipment). The overall infection rate of laboratory confirmed SARS-CoV-2 among HCWs in the department of gastroenterology was 4.8% (10/209).

**Table 1.** Infection, personal protection, and screening of HCWs with suspected and confirmed COVID-19

	HCW 1	HCW 2	HCW 3	HCW 4	HCW 5	HCW 6	HCW 7	HCW 8	HCW 9	HCW 10	HCW 11	HCW 12	Mean ±SD	%
Sex	Female	Female	Female	Female	Female	Female	Female	Female	Female	Female	Female	Female		
Age (y)	32	28	38	31	25	33	45	26	37	30	39	49	34.4 ± 7.4	
Occupation	Nurse	Nurse	Nurse	Nurse	Nurse	Nurse	Nurse	Nurse	Nurse	Nurse	Nurse	Nurse		
History of allergy	No	Yes	Yes	Yes	No	Yes	No	No	No	Yes	No	Yes		50
History of illness	No	No	No	No	No	No	No	No	No	No	No	No		
Infection														
Close contact (<1 m)														
Life nursing	+	+	+	-	-	-	-	-	-	-	+	-		33.3
Ward rounds	+	+	+	-	-	-	-	-	-	-	-	+		33.3
Physical examination	+	+	+	-	-	+	-	-	-	-	+	-		41.7
Sampling	+	-	-	-	-	-	-	+	-	+	-	-		25.0
Injection	+	+	+	+	-	-	+	+	-	+	+	-		66.7
Endoscopy	-	-	-	+	-	-	-	-	+	-	-	-		16.7
Cleaning	+	-	-	+	-	+	-	-	-	-	+	-		33.3
Talk without masks	+	-	-	-	+	+	-	+	+	-	-	+		
PPE														
White gown	+	+	+	+	+	+	+	+	+	+	+	+		100
Surgical masks	+	+	+	+	+	+	+	+	+	-	+	+		91.7
N95 masks	-	-	-	-	-	+	-	-	-	+	-	-		16.7
Hats	-	+	+	+	+	+	+	-	+	+	+	-		75
Gloves	-	+	+	+	-	+	+	+	+	+	-	-		75
Goggles	-	-	-	-	-	+	-	-	-	+	-	-		16.7
Isolation clothing	-	+	-	-	-	+	-	-	-	+	-	-		25
Medical protective clothing	-	-	-	-	-	+	-	-	-	+	-	-		16.7
Shoe covers	-	-	-	-	-	+	-	-	-	+	-	-		16.7
Facial shield	-	-	-	-	-	+	-	-	-	+	-	-		16.7
Comprehensive respirator	-	-	-	-	-	-	-	-	-	-	-	-		0
Hand hygiene	Usu	Usu	Alw	Usu	Alw	Usu	Alw	Usu	Usu	Alw	Alw	Alw		
Duration of exposure (d)	3	7	1	1	1	NA	2	5	1	10	2	NA	3.3 ± 3.1	
Average time per contact (min)	6	4	3	10	25	NA	5	5	20	NA	4	NA	8.7 ± 8.1	
Estimated individual contacts	10	24	4	2	1	NA	8	20	3	NA	6	NA	9.1 ± 7.9	
Interval 1 (d) ^a^	2	6	6	7	10	NA	10	20	7	9	7	NA	8.4 ± 4.7	
Examination														
Interval 2 (d) ^b^	3	0	0	11	0	0	0	0	5	2	0	0	1.8 ± 3.3	
Results of chest CT scan	+	+	+	+	+	-	+	-	+	+	+	+		83.3
Total chest CT scans	4	5	5	2	2	5	3	2	2	2	4	3		
Results of nucleic acid/antibodies tests	+	+	+	+	+	+	+	+	+^c^	+^c^	-	-		66.7
Sampling														
Nasopharyngeal swab	0	4	4	4	0	1	0	2	0	3	4	1	1.9 ± 1.8	
Throat swab	3	1	1	1	3	5	4	1	1	0	0	2	1.8 ± 1.6	
Total	3	5	5	5	3	6	4	3	1	3	4	3	3.8 ± 1.4	

Abbreviations: Alw, always; CT, computed tomography; HCW, health-care worker; NA, not available; PPE, personal protective equipment; Usa, usually.

a Interval between exposure and symptom onset.

b Interval between symptom onset and first CT scan.

c Positive result of antibodies test and negative result of nucleic tests.

The infection was clustered from January 20 to February 10, 2020. These HCWs worked for 7 h on a weekday and a nightshift every 6 d as usual. There were 4 (33.3%) HCWs having contact with patient 1 and 9 (75%) HCWs with patient 2. Close health-care behaviors (distance less than 1 m) contained assessment of vital signs and physical examination (6/12; 50%), life nursing (4/12; 33.3%), ward rounds (4/12; 33.3%), endoscopic examination (2/12; 16.7%), cleaning patients’ vomitus and stuff (4/12; 33.3%). Although median duration of exposure was 2 d (interquartile range [IQR], 1.0-4.5), time of each contact was short (8.1 min ± 5.6 min). The total contacts of each HCW with suspected COVID-19 cases fluctuated from 1 contact to 24 contacts (9.1 ± 7.9 contacts). Most of them wore white/blue gowns (12/12; 100%), medical surgical masks (11/12; 91.7%), disposable hats (9/12; 75%), gloves (7/12; 58.3%), and isolation clothing (3/12; 25%) before abnormal chest CT images were found in the 2 patients (Table 1). Two (16.7%) HCWs even wore N95 masks and medical protective clothing when providing medical care for patients. All HCWs have performed hand hygiene more often than before. Three (25%) HCWs provided that they talked with colleagues without masks and had meals in the same room. The median incubation periods were 7 d (IQR 6.3-9.8). There was no familial cluster or household infection of COVID-19 in these 12 ill HCWs.

### Screening of HCWs With Suspected or Confirmed COVID-19

Due to close contact with suspected patients, a rapid screening was made once any HCW presented with respiratory symptoms. All ill HCWs were female nurses with median 32.5 y of age (IQR 29.5-38.3), who had mostly been working for over 10 y and in good health condition (Table 1). Chest CT scans were performed in 8 HCWs on the same day of onset (Figure 1). Of 10 HCWs with confirmed COVID-19, 30% (7/10) HCWs were categorized into the general type of COVID-19, and 20% (2/10) HCWs belonged to the mild type of COVID-19. There was only 1 HCW with severe COVID-19 found to have oxygen saturation < 93%.

The symptoms onset among HCWs were fever (4/12; 33.3%), dry cough (5/12; 41.7%), fatigue (3/12; 25%), running nose (3/12; 25%), myalgia (3/12; 25%), and pharyngalgia (2/12; 16.7%) (Table 2). It is noteworthy that 2 HCWs with confirmed COVID-19 had no symptoms; they took chest CT scans because of close contacts with suspected cases. Three HCWs with confirmed COVID-19 were hospitalized (Figure 1), and lymphocytopenia was found in 2 HCWs.

**Table 2.** Symptom onset and recovery of HCWs with suspected and confirmed COVID-19

	HCW 1	HCW 2	HCW 3	HCW 4	HCW 5	HCW 6	HCW 7	HCW 8	HCW 9	HCW 10	HCW 11	HCW 12	Mean ±SD	%
Symptom onset														
Fever	+	+	-	+	-	-	-	-	+	-	-	-		33.3
Cough	-	-	+	+	-	+	-	-	-	+	-	+		41.7
Fatigue	+	-	-	+	-	-	-	-	+	-	-	-		25
Running rose	-	-	-	+	-	-	-	-	-	+	+	-		25
Myalgia	-	-	-	+	-	-	-	-	+	-	+	-		25
Pharyngalgia	-	-	-	+	-	-	+	-	-	-	-	-		16.7
Recovery														
Interval 3 (d)^a^	23	10	37	16		32	12		30	21	7	21	20.9±9.9	
Interval 4 (d)^b^	11	5	17	15	10		5		30	9	31	10	12.4±7.7	
Interval 5 (d)^c^	10	11	14	21	7	6	5	4					9.8±5.7	

Abbreviation: HCW, health-care worker.

a Interval between symptom onset and away.

b Interval between symptom onset and computed tomography (CT) improvement.

c Interval between symptom onset and nucleic acid transfer.

Chest CT images of viral pneumonia were shown in 10 HCWs. Briefly, GGO and patchy shadows were seen in lungs of 8 (80%) HCWs and 5 (50%) HCWs, respectively. Lesions of lungs were relatively milder, smaller, and more focused in HCWs with COVID-19. Baseline and follow-up chest CT did not show any abnormalities in 2 HCWs (16.7%).

### Quarantine and Recovery of HCWs With Suspected or Confirmed COVID-19

All HCWs were quarantined at the early stage, mostly on the same day of symptom onset. The isolation locations were home, designated hotels, Fangcang Shelter hospitals, or hospitals assigned for COVID-19 treatment (Figure 1). Owing to the early recognition and quarantine of source patients and HCWs with COVID-19, as well as more strict surveillance, there was no more infections in the department of gastroenterology.

All patients recovered after medicine and supportive treatment. Lesions of lungs were absorbed within 5 d to 31 d (12.4 d ± 7.7 d) in 10 HCWs who obtained imaging features of viral pneumonia (Table 2). Clinical symptoms improved markedly around 1 wk (8.1 d ± 4.8 d). It required average 9.8 d and 20.9 d to obtain viral shedding of SARS-CoV-2 RNA and clinical cure, respectively (Table 2). There was no recurrence during follow-up.

### Psychological Stress of the Outbreak on Ill HCWs in a Nonfrontline Department

**Figure 2.**
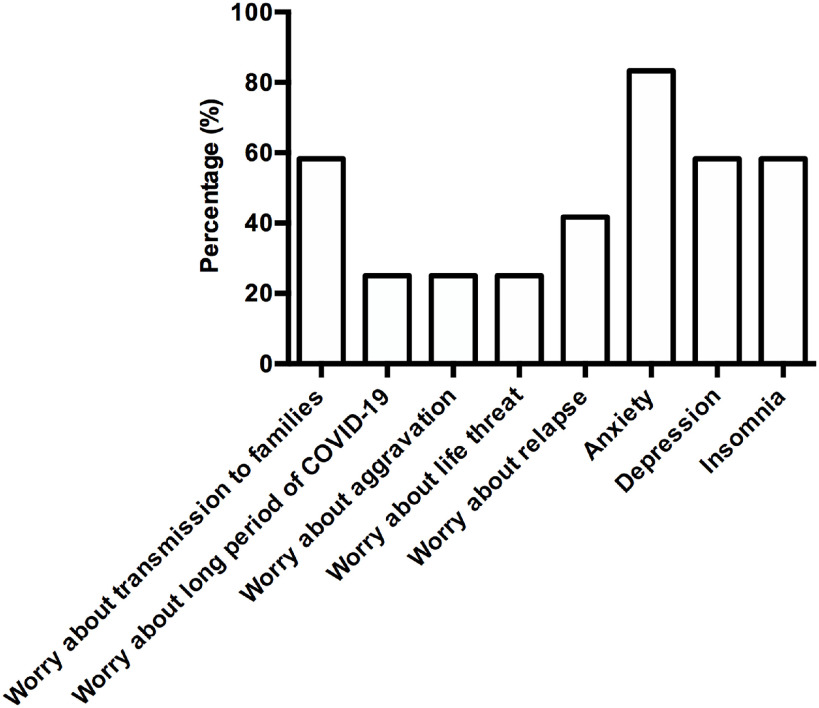
Psychological effects of the outbreak on Ill HCWs in a department.

The online questionnaire survey showed that half of 12 (50%) HCWs considered COVID-19 as influenza before SARS-CoV-2 infection. However, 3 (25%) HCWs worried that COVID-19 would be long lasting. Three (25%) HCWs worried about aggressive, even life-threatening, disease upon infection (Figure 2). After SARS-CoV-2 infection, half of 12 (50%) HCWs still considered COVID-19 as influenza, and believed that they will recover soon. Five (41.7%) HCWs were afraid of recurrence or more aggressive disease. Although no one worried about persistently detectable SARS-CoV-2 RNA after clinical recovery, 7 (58.3%) ill HCWs still worried about transmission of SARS-CoV-2 to families.

Other subjective psychological feelings included anxiety (10/12; 83.3%), depression (7/12; 58.3%), and insomnia (7/12; 58.3%) accompanying with dreaminess (Figure 2). According to their own subjective feelings, the proportion of HCWs reporting a severe degree of anxiety, depression, or insomnia was relatively low (8.3%, 0, 8.3%, respectively). A total of 25% (3/12) ill HCWs thought that they might lose confidence and enthusiasm in medical work. However, most (10/12) infected HCWs demonstrated they would always keep strengthening awareness of personnel professional risk and self-protection in the future clinical practice.

## Discussion

Isolation of patients and contact tracing are key tools for controling outbreaks of infectious diseases, especially for hospital-based infection. We present an intact and comprehensive study of in-depth epidemiological surveillance, screening, and follow-up of HCWs infected with SARS-CoV-2 in a nonfrontline clinical department from Wuhan City. To our best knowledge, there were few studies focusing on SARS-CoV-2 infection of HCWs outside the frontline department in health-care settings. We found patients with COVID-19 might first seek treatment for digestive problems and be a source of nosocomial infection to HCWs and other patients. Our study demonstrated that close contact and inadequate personal protection were major risk factors of SARS-CoV-2 infection. Early screening and quarantine of COVID-19 cases were effective measures to cut off virus spreading at the health-care facility. Most HCWs with COVID-19 in our study experienced a mild clinical course and good recovery, while generally having psychological stress. These findings can not only contribute to better understanding of human-to-human transmission among HCWs, but also provide informative suggestions for prevention and control during the COVID-19 outbreak.

In our study, 2 source patients were related to primary or secondary infection of 10 HCWs. When facing an emerging outbreak with extremely limited knowledge, HCWs with occupational exposure are at great risk of infection, especially at the beginning of the outbreak. During the SARS outbreak, HCWs consisted of 22% infected patients and one-third of deaths.^13^ COVID-19 is a new occupational disease. In a hospital in Wuhan, 40 HCWs among 138 patients were reported to be infected with SARS-CoV-2. In a surgical department, 1 patient initially with abdominal symptoms possibly infected more than 10 HCWs and was possibly a super spreader.^7^ Analysis of early transmission dynamics demonstrated the infection rate of HCWs increased from 3% (January 1, 2020 to January 11, 2020) to 7% (January 12, 2020 to January 22, 2020),^14^ which was up to 11% in Italy on April 10 and 19% in America on April 9, 2020, based on available data.^11,12^ In China, 88% of infected HCWs were reported from Hubei Province. The majority of HCWs were infected with SARS-CoV-2 in the first 2 months of the outbreak under insufficient self-awareness and personal protective equipment (PPE), especially in late January 2020. An epidemiological survey inferred that approximately 40-55% of infections in HCWs were occupationally exposed, whereas others were probably community-involved.^8,11,15^

In the early stage of the outbreak, occupational protection had been reinforced compared with that before the outbreak. At least all HCWs in the clinical department wore surgical masks or N95 masks when providing medical care. Hand hygiene was performed more often with chlorine-containing disinfectants. The possible reasons contributing to infection of HCWs were as following: (1) suspected patients did not wear masks; (2) disinfecting of surfaces was insufficient; (3) hand hygiene was dismissed sometimes or not standardly performed each time; (4) PPE was inadequate if with only surgical masks and gowns, some HCWs were even talking and having meals together in 1 dining room without masks; (5) taking off PPE was not according to specifications. There were rare studies exploring times and duration of contacts to acquire COVID-19. In a report on 30 HCWs infected with SARS-CoV-2, their average number of close contacts with COVID-19 patients was 12 and cumulative contact time was 2 h.^16^ Number of contacts and estimated duration of contacts with source patients were fewer and shorter, respectively, in infected HCWs in our study. The infection rate was 40% in all HCWs who came in contact with source patients, indicating the high infectivity of SARS-CoV-2. The severity of illness in HCWs with COVID-19 was associated with inadequate prevention and protection, longer duration, and more contacts with patients.^16^ Therefore, contact, droplet, and airborne precautions should be applied in health-care settings according to risk levels.^17^

Symptoms, including fever, dry cough, myalgia, and fatigue, were used to screen the potential infection of HCWs.^6,7^ However, only 4 (33.3%) infected HCWs had a fever in our study, far below that of current reports.^7^ In addition, alert should be raised that there are asymptomatic infections, as 2 HCWs (16.7%) with SARS-CoV-2 infection did not have any symptoms in our report; 7% asymptomatic infection was also reported by Xu et al.^18^ The 1 with asymptomatic infection might be a potential source of infection. To reduce the transmission to colleagues and vulnerable patients, once HCWs are found with clear evidence of exposure or similar clinical manifestations of COVID-19, the nucleic acid test, antibodies test of SARS-CoV-2, and chest CT scan are strongly recommended to screen for SARS-CoV-2 infection.

Facing this global infectious public health event, HCWs are under both physical and psychological pressure. The ever-increasing infected cases, huge workload, shortage of PPE, widespread media coverage, lack of specific drugs, and feelings of being inadequately supported may all contribute to the mental burden.^19^ A survey completed among 1257 HCWs showed nurses, women, frontline HCWs, and those working in Wuhan, China, revealed that these persons exhibited more severe impacts on mental health than other HCWs.^19^ However, another study demonstrated that HCWs in the nonfrontline clinical department may suffer much stress and burnout, even than those on the frontline,^20^ because HCWs on the frontline may have more professional knowledge to address infectious patients and feel a greater sense of control of their situation with well-prepared PPE. Worry, anxiety, and insomnia were the main mental discomforts reported in our study, which was consistent with previous research.^20,21^

There are some limitations. First, it was subject to recall bias, as it was based on the self-reports by HCWs. Second, this was a retrospectively single-center study with small samples. Third, the comparison in exposures was not designed in HCWs with or without COVID-19 caring for source patients, for HCWs without COVID-19 failed to recall the contact details. Fourth, the psychological stress was based on the subjective feeling of the HCWs, but not evaluated by special validated objective scales.

## Conclusions

Health-care systems will be overwhelmed for a long time fighting the COVID-19 outbreak. There are increasing infection risks and psychological stress for HCWs because of close contact with COVID-19 patients under insufficient protection conditions. Sufficient precautionary measures, early screening and quarantine, and active psychological screening regarding COVID-19 are urgently required for HCWs.
